# The Sympathetic Nervous System in Dental Implantology

**DOI:** 10.3390/jcm12082907

**Published:** 2023-04-17

**Authors:** Johannes Raphael Kupka, Keyvan Sagheb, Bilal Al-Nawas, Eik Schiegnitz

**Affiliations:** Department of Oral and Maxillofacial Surgery, University Medical Center Mainz, 55131 Mainz, Germany

**Keywords:** sympathetic nervous system, adrenoceptor, dental implantology, oral surgery

## Abstract

The sympathetic nervous system plays a vital role in various regulatory mechanisms. These include the well-known fight-or-flight response but also, for example, the processing of external stressors. In addition to many other tissues, the sympathetic nervous system influences bone metabolism. This effect could be highly relevant concerning osseointegration, which is responsible for the long-term success of dental implants. Accordingly, this review aims to summarize the current literature on this topic and to reveal future research perspectives. One in vitro study showed differences in mRNA expression of adrenoceptors cultured on implant surfaces. In vivo, sympathectomy impaired osseointegration in mice, while electrical stimulation of the sympathetic nerves promoted it. As expected, the beta-blocker propranolol improves histological implant parameters and micro-CT measurements. Overall, the present data are considered heterogeneous. However, the available publications reveal the potential for future research and development in dental implantology, which helps to introduce new therapeutic strategies and identify risk factors for dental implant failure.

## 1. Introduction

Improvement of osseointegration, biological compatibility and reduction of morbidity are intense research topics in implant dentistry. New technologies, materials and insights into the mechanisms underlying failure and success should help to address these challenges [1,2].

There was an enormous increase in the prevalence of dental implants between 1999 and 2016 in the US in individuals aged between 55 and 64 years by ~1000% [3]. The technology has revolutionized the possibilities of tooth replacement and created a new dimension compared to conventional fixed and removable prosthetics [4,5,6]. The direct anchorage in the bone demonstrates a noticeable difference for the patients and significantly improves their quality of life [7,8].

The mechanism responsible for the stability of the construction and its outstanding biomechanical properties is the so-called osseointegration [9]. It has already been studied and described many times [10,11,12] but, even nowadays, not all the intricacies of this process are understood [13]. Osseointegration is defined as the biomechanical connection between the implant surface and bone. It represents a structural, as well as functional, connection [14]. In the initial phase, the mechanical anchoring of the thread in the jawbone is the base for the stability of dental implants. In the course of remodelling processes, osseointegration occurs, which is responsible for the secondary stability [15].

Implant placement is foremost an injury to the bone, and the reaction to this process is similar to that of a fracture [16]. First, a blood clot is formed by fibrin polymerization. It serves as a basis for the sprouting of vessels and extracellular matrix formation [17]. Subsequently, cells responsible for the synthesis of new bone migrate into the gap surrounding the implant. The subsequent bone accretion can occur from two directions: from cells that have settled on the implant surface as contact osteogenesis and from the bone surface itself as distance osteogenesis [18].

The initially formed disorganized woven bone is now further remodeled to distribute occlusal loads [19]. At the center of this sequence of degradation, formation, and remodeling are the two most important cell types of the bone: osteoclasts for bone resorption and osteoblasts for bone formation. They ensure a lifelong balance and adaptation in the entire skeletal system and, thereby, around dental implants [20].

The view on osseointegration as fracture healing alone explains some physiological processes, but research shows that there are other aspects to consider that add to the complexity. For example, titanium or titanium oxide are not bioinert [21]. They induce a foreign body reaction, which shields the human body as much as possible from the foreign material “implant” [22]. A dense bone layer results from this inflammatory reaction. However, this also provides stability [23]. Although many aspects of osseointegration are already understood, some influencing factors remain unclear. Future work in preclinical research will help to understand osseointegration and apply findings to the clinical phenomenon of secondary stability [13].

Stress and mental strain are a severe burden of today’s society [24], which affects practitioners and patients [25,26]. The physiological processes underlying the effects of stress are multifaceted, and current research is attempting to approach them in several ways [27,28]. One aspect is the sympathetic nervous system (SNS) [25]. Together with the parasympathetic nervous system, it forms the major components of the autonomic nervous system [29]. The SNS mediates the fight-or-flight response, while the parasympathetic nervous system is active during periods of rest [29]. However, recent research has shown that the range of action, in particular of the SNS, extends much further [30]. Its central neurotransmitter, norepinephrine (NE), mediates an influence on a wide variety of tissues [31]. NE acts via the so-called adrenoceptors (ARs) that trigger mostly G-protein-mediated signaling cascades. Several different effects are achieved depending on the receptor subtype [32]. Hamano et al. showed that AR expression on periodontal fibroblasts depends on occlusal forces, and they probably play a role in homeostasis [33].

Phases of healing and remodeling prolong the treatment time until the patient receives the finished prosthesis. Efforts to shorten it, for example, by immediate implant placement and loading, are not always practical and sometimes show reduced success rates [34]. Furthermore, long-term stability also depends on sufficient osseointegration [35]. For this reason, current research in dental implantology aims to accelerate and improve osseointegration. For example, several authors have investigated the influence of bone morphogenetic protein (BMP) or various bioactive surface modifications [36,37]. However, our knowledge about the autonomic nervous system in osseointegration needs an update. Such an influence on bone remodeling would be highly relevant, especially since more than 150 million people in Europe suffer from hypertension, and depression has a prevalence of 18% [38,39]. Drugs that influence the SNS are of great value for both diseases. These include beta-blockers, tricyclic antidepressants (TCAs) and serotonin-norepinephrine reuptake inhibitors (SNRIs) [40].

Thus, this review aims to summarize the current state of basic research in dental implantology on the sympathetic nervous system and to link it with findings from other fields. It will identify future research needs and open up new perspectives. It serves as a basis for a central aspect of scientific and clinical research in dental implantology.

## 2. Methods

This review was guided by the PRISMA checklist and statement and was registered in the International Prospective Register of Ongoing Systematic Reviews (PROSPERO) (ID: 389222). We wanted to clarify the following questions:

− What influence does the SNS have on bone metabolism?
− How does the SNS influence the osseointegration of dental implants?
− What are the underlying mechanisms?
− What is the role of drugs whose target is the SNS?

Suitable PICO criteria were defined for this purpose:

-P: Animals with dental implant surgery

-I: Influencing the sympathetic nervous system through an applied intervention or medication

-C: Implant placement without any additional treatment regarding the SNS

-O: Histological, biomechanical, and radiographic measurements

A systematic electronic literature search was performed in the databases Medline (PubMed), Cochrane and Web of Science. The reference list and citations were also searched for relevant studies. The last check was performed on 17 August 2022. The search was documented using commercially available spreadsheet software (Microsoft Excel). Using the citation software Endnote 20, the results were collected, and duplicates and triplicates were excluded.

J.K. performed the initial search independently. E.S. subsequently controlled it. After duplicate exclusion, J.K. determined the study eligibility based on the title and abstract. If there was disagreement with the other authors, consensus was reached in a joint discussion. Table 1 provides an overview of the search terms used.

One of the aims of this review was to provide a comprehensive overview of the topic in basic research. For this reason, a wide range of study types was accepted only in that field. Clinical studies, such as observational studies, randomized controlled trials, controlled and uncontrolled trials, systematic reviews with and without meta-analyses, longitudinal studies, case studies and series, were excluded. On the other hand, all non-clinical studies, such as laboratory research, animal studies, in vitro or ex vivo studies, or post-mortem studies, were suitable. The animal studies were rated using the SYRCLE’s tool for assessing the risk of bias [41]. There were no further restrictions regarding the type or the date of the publication, in order not to miss any results. However, only literature in the German and English languages was included. Under these conditions, all subheadings and MESH terms were searched, as well as the title and abstract.

Only studies that concerned dental implantology and the influence of the sympathetic nervous system through an applied intervention or medication or investigated relevant structures of the SNS were included. The broad field of search terms explains the extent of results that could be excluded with ease because they did not thematically correspond in any way to the aim of this review.

## 3. Results

**Figure 1:**
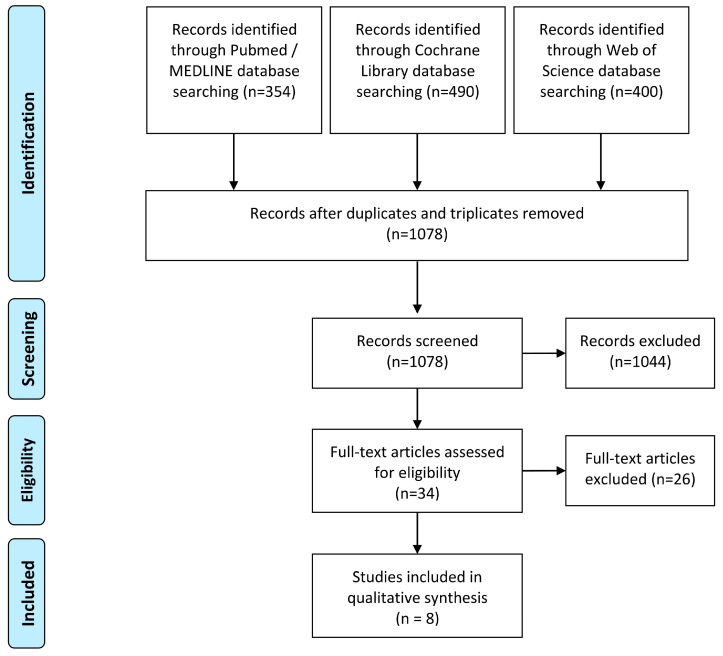
PRISMA flow diagram.

The first search yielded 1244 results. These could be reduced to 1078 after the exclusion of duplicates. Since the search terms were very general, many publications were thematically far from the desired results (Figure 1). In the end, 34 results remained, whose full text was examined for suitability. 

Five studies were excluded because they were related to stress and anxiety during surgery [42,43,44,45,46]; five were excluded because they were related to anesthesia [47,48,49,50,51]; and four were excluded because they studied the influence of the SNS on orthodontics [52,53,54,55]. Four publications dealt with the effect of the SNS on dental pulp [56,57,58,59]. The systemic effect of the SNS was the main focus of three publications [60,61,62]. Others were clinical studies and therefore excluded [63,64,65,66,67].

Eight studies remained that fulfilled the inclusion criteria [68,69,70,71,72,73,74,75]. Five of them dealt with the effects of propranolol [69,70,71,72,73].

**Figure 2:**
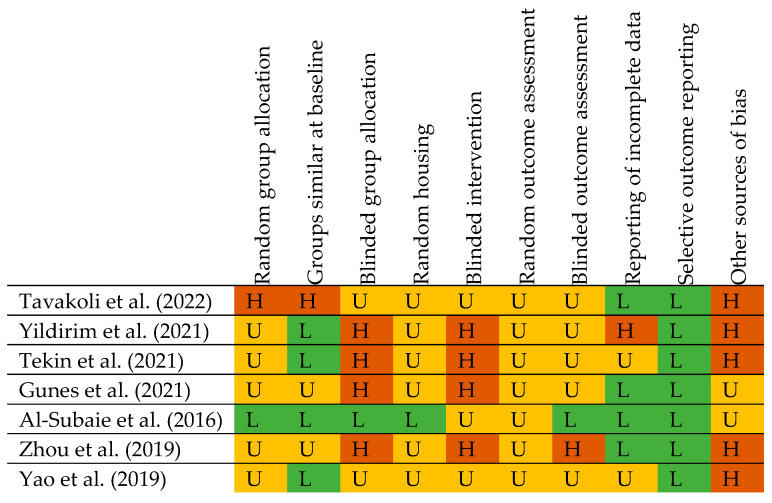
Risk of bias assessment according to the SYRCLE risk of bias tool [41]. H: High-risk; L: Low-risk; U: Unclear.

The studies by Tekin et al. and Zhou et al. showed a high risk of bias in four out of ten categories, while Al-Subaie et al. had a low risk of bias in seven categories (three were unclear) [71,73,74]. The high number of unclear items is not unusual, as Faggion et al. showed in their review about the risk of bias in animal experiments in implant dentistry (see also Figure 2) [76].

The results of the eight included studies are now briefly presented (see also Table 2).

Morinaga et al. investigated the influence of the Neuronal PAS domain protein 2 on osseointegration [68]. Therefore, most of this detailed publication is not part of this review. However, a final section dealt with the consideration of molecular mechanisms. A chemical gene analysis was performed with bone marrow-derived stem cells (BMSCs), cultured on simulated implant surfaces. Evidence was found that α2-receptors and downstream signaling pathways via cAMP and CREB may be involved. The expression of adrenoceptor subtypes on the mRNA level showed an upregulation of α2- and β1-receptors upon contact with rough implant surfaces compared to polystyrene and machined surfaces. The expression of beta 2 receptors remained unchanged. These are the only in vitro results, which are included.

The most recent study by Tavakoli et al. investigated the effect of propranolol on dental implants in four street dogs with three implants each [69]. Two dogs received oral propranolol after tooth extraction and continued to receive it after implant placement. Two dogs served as a control group without effective medication. The bone implant contact (BIC) was examined histologically by removing the implants with the surrounding bone through a trephine drill. After 4 weeks, there was a significant difference in favor of the test group (68.33% vs. 20.22%, *p* = 0.002), but this was not present after 9 weeks (68.60% vs. 50.17%, *p* = 0.096).

Three publications were prepared by a research group at Firat University [70,71,72]. They are conceptually similar and studied the effect of propranolol on osseointegration in rats.

Tekin et al. did not find a significant difference in biomechanical testing with the reverse torque test [71]. However, alkaline phosphatase in blood samples was increased in rats treated with propranolol.

Gunes et al. additionally used a bovine bone graft. However, no significant differences were found in the histological parameter of newly formed bone tissue to the grafted area and the blood values [72].

Yildirim et al. found no differences in blood values, but a significantly higher BIC ratio was noted by histologic analysis [70].

The fifth study on the effect of propranolol on dental implants in rats by Al-Subaie et al. did not use realistic dental implants, but simulated them using titanium cylinders [73]. With a larger hole on one side, a bone defect was simulated. Micro-CT examination revealed significantly smaller remaining defects for the propranolol-treated animals (1.67 ± 0.35 mm^3^ vs. 2.04 ± 0.29 mm^3^, *p* < 0.001). The ratio of bone volume to trabecular volume also increased. Histological analysis showed fewer osteoclasts on the surface of the bone defects. The amount of mineralized tissue and collagen increased. As in the study of Yildirim et al., BIC was assessed and consistently increased in the test group.

Zhou et al. did not want to block the SNS, but to promote its effect [74]. For this purpose, they inserted 14 self-made implants in eight beagles after they removed the upper lateral incisors in the sense of immediate implantation. After 1 week, four dogs received daily electrical stimulation at the sympathetic cervical ganglion. This procedure continued for 3 weeks. Then, the animals were sacrificed and micro-CT images were obtained, as well as histological examinations.

The electrical stimulation showed only slight effects on the cardiovascular system. However, it resulted in greater new bone formation: after 4 weeks, a gap between bone and implant was still detectable in the control group; in the test group, it had largely healed. Bone mineral density increased significantly in the stimulated group after 4 weeks (0.62 ± 0.05 vs. 0.47 ± 0.07). Periodontal and peri-implant bone indices showed no differences after electrical stimulation. Thus, pre-existing bone was not affected.

Harvested primary osteoblasts and sympathetic neurons were isolated from the rats and cultured separately or together. The survival rate of osteoblasts was higher in coculture with sympathetic neurons. Microscopy showed direct communication of neurons with osteoblasts.

Yao et al. compared n = 20 chemically sympathectomized mice with n = 20 others as a control group [75]. In all of them, an implant was placed in the femur. The measured blood levels for osteocalcin and CTX-I were significantly different at weeks 2 and 4 after surgery. Osteocalcin decreased in the control group and increased in the sympathectomy group. CTX-I increased by approximately 150% in the control group, whereas it increased by only about 5% in the sympathectomy group.

Micro-CT examinations showed significant differences for all measured values after 4 weeks. The quotient of bone volume and total volume, the proportion of osseointegrated implant surface, and the average thickness and number of trabeculae were reduced in the sympathectomy group. The quotient of bone surface area and bone volume, as well as the average distance of the trabeculae, increased.

Histological examination showed no significant difference in bone implant contact ratio at week 2, whereas, after 4 weeks, BIC increased by only 43.6% in test animals and 71% in the control group. The measured mineral apposition rate and the bone formation rate per bone surface indicated an impairment of osseointegration by sympathectomy.

A push-in test was performed to test the biomechanical properties. The bone implant integration strength was 37% higher in the control group.

## 4. Discussion

### 4.1. What Influence Does the SNA Have on Bone Metabolism?

Bone remodeling is a central part of osseointegration. Some researchers investigated the influence of the sympathetic nervous system on bone. The autonomic nervous system is roughly divided into the sympathetic and parasympathetic nervous system [29]. The SNS mediates its effect via the neurotransmitter norepinephrine, which is synthesized in the neurons with the help of the key enzyme tyrosine hydroxylase, and then packaged in vesicles and released in response to an appropriate signal [78]. The vesicles also contain the cotransmitters ATP and NPY, which can modify the effect on the target tissue [79]. Two enzymes are responsible for the degradation of NE: monoamine oxidase and catechol-O-methyltransferase. Another mechanism for terminating the signal is reuptake via the norepinephrine transporter [80]. These mechanisms are used as targets of common pharmaceuticals [81].

The fibers of the SNS innervate various tissues [31,82]. The best-known effects include bronchodilatation, a positive chronotropic effect on the heart, or mydriasis. These are known as the fight-or-flight response [83]. Although it is not necessary to run away from a potential predator in our modern society, via regulatory circuits in the central nervous system, specifically in the brainstem and hypothalamus, the sympathetic nervous system is involved in the processing of and responses to external stressors [84,85]. For example, an increased stress level can be detected by elevated norepinephrine levels in the blood [86], and even the immune system is controlled by the SNS (although this aspect is certainly very relevant for future research on osseointegration, it is not part of this review due to the current data situation) [87,88].

In bone, sympathetic nerve fibers could also be detected immunohistochemically via TH, dopamine beta-hydroxylase (another enzyme in NE synthesis), and neuropeptide Y [89]. They are present in the bone marrow, the periosteum and the compacta. Many of them are associated with blood vessels in Havers and Volkmann channels. The exact distribution is still partly unexplored [90,91,92,93]. However, it has become clear that areas exposed to higher mechanical loading also have higher fiber density [94]. Functional analyses pose a problem in addition to these anatomical studies. In contrast to concentration measurements in the synovial fluid, detection of the outflow is hardly possible in the bone, due to its nature [95].

Finally, α- and β-adrenoceptors (ARs) mediate the effect of NE at the target tissue. These heptahelical G-protein-coupled receptors trigger different signaling cascades intracellularly [96].

α1-ARs can be further divided into three subgroups A, B, and D. All are G_q_ coupled, so via activation of phospholipase C, Phosphatidylinositol 4,5-bisphosphate is cleaved into inositol trisphosphate and diacylglycerol. An increase in intracellular calcium concentration occurs and mediates, for example, the contraction of smooth muscle cells [97]. The α1-ARs are most sensitive to NE and less sensitive to epinephrine [80]. α1B- and α1D-receptors have been detected on human osteoblasts [98,99] and α1B on osteoclasts [100]. However, their function has not yet been definitively elucidated. In vitro, the α1-agonist cirazoline led to higher proliferation rates of human osteoblasts [98].

α2-ARs, in contrast, are presynaptic receptors that are G_i/o_ coupled. All three subgroups (A, B, C) relay the signal via inhibition of adenylate cyclase [101]. In part, the relevance to bone metabolism is only indirect, manifested by regulating the NE outflow [102,103]. Others detected the mRNA of α2-ARs in both osteoclasts and osteoblasts. Although lower bone mass would be expected in α2A and α2C knock-out mice due to NE excess (see below), the opposite occurred [102]. Again, further studies are needed.

It is believed that β-receptors play the most significant role [30]. There are also three subtypes: β1-Ars are typically found in the heart and increase heart rate and contractability [104]. β2-ARs are present in many tissues, but are mostly known for vaso- and bronchodilatation [105]. β3-ARs are controlling the thermogenesis in brown adipose tissue [106]. They are all G_s_-coupled and increase the cyclic adenosine monophosphate (cAMP) concentration in the cell via the activation of the adenylate cyclase. cAMP binds the regulatory subunit of protein kinase A (PKA), which can then phosphorylate and regulate enzymes [107]. β1- and β2-receptors could be detected on osteoblasts and osteoclast-like cells in humans, whereby β2 was more common [108,109,110]. Stimulation of this receptor results in the expression of RANKL on osteoblasts [111]. RANKL binds to RANK on the surface of osteoclast precursors and induces their fusion and differentiation. The direct effect of β-ARs on osteoclasts is the stimulation of osteoclast differentiation and activation of bone resorption [100]. These results were confirmed in vivo, where treatment with β-agonists showed a catabolic effect on the bone [112].

The comparison of β2-, β1- and β1/2-deficient mice suggests that β1 and β2-ARs cause different effects on bone turnover: as expected, β2-deficient mice exhibited a high bone mass phenotype, whereas mice missing β1-AR, as well as β1/2, showed a lower bone mass [113].

Overall, the dominant effect of the SNS on bone via α- and β-adrenoceptors is likely to be catabolic. Further studies will be necessary to investigate the interplay of different cell types and receptors, particularly in humans.

### 4.2. Included Studies

Preclinical research offers the opportunity for extensive standardization, even of the test subjects, which is not achievable in clinical studies [114]. Since the present data are relatively heterogenous, the central message of each included study will be discussed separately. Arguments on whether the SNS promotes or impairs osseointegration will be collected and clarified. Points, once mentioned, apply analogously to considerations of the other publications, where appropriate.

Although Morinaga et al. did not primarily focus on adrenoceptors, it is a study with some relevance to this review [68]. They linked ARs to a mechanism that is likely related to improved osteointegration. However, the data are still weak and need further investigation to support the thesis [68,115].

Indeed, that BMSCs express ARs is widely known and proven. Hedderich et al. showed, in a high-quality study, that the proliferation of BMSCs is affected by NE through the PKA and ERK1/2 signaling pathway [116]. However, the dependence of expression on the culture surface is a new factor. Future studies should always use implants that are as realistic as possible. Schmitt et al. showed that the effect of BMP depends on the surface on which BMSCs grow [117]. Morinaga’s work may bring the even less noticed α2-receptor into focus. Mlakar et al. have shown the presence of the α2A-receptor on osteoblasts, and their results suggest a promoting influence on bone resorption [103].

The following five studies focused on the effect of propranolol and provided a first answer to the fourth question mentioned in the methods section.

The results of Tavakoli et al. suggest that propranolol has a favorable effect on bone metabolism, especially in the first phase [69]. The strengths here lie in the use of conventional implants, as well as in the use of dogs. They are preferable as experimental animals for dental implant research: the implants can be inserted intraorally; dogs provide adequate jaw size to allow standard surgical access; and their jaw movements are not designed for plant-based diets, which would result in atypical loading [118]. The disadvantages of this study are the small group size (n = 2 for each group) and the inhomogeneity due to the use of street dogs. Standardization is essential in basic research to detect possible isolated effects. Thus, the design alone lowers the value of the study [114].

Regarding the publications from Firat University, the first item to discuss is the choice of the experimental animal [70,71,72]. Rats are cheaper and faster to breed but inferior to dogs, due to the above-mentioned factors [119]. The reverse torque test is a widely established test for animal studies, which has also been used in humans. Because of the potential damage to the implant–bone interface, it is no longer in use [120]. Newly healed implants have not yet aligned their bone structure based on loading. Rotational motion-exerting shear forces on the bone is atypical and, compared to traction and compression, the bone is least able to resist [121].

The difference found in alkaline phosphatase concentration, a classic parameter of bone metabolism, must be critically questioned because it has a lower specificity compared to modern bone remodeling values [122]. Calcium and phosphate are also very unspecific, and changes are based on many other factors, such as pH. The International Federation of Clinical Chemistry (IFCC) has recommended, in a position paper, the use of CTX-I and PINP as preferred bone resorption and formation markers—especially for studies [122].

Apart from propranolol, further work is necessary using specific agonists or antagonists at the corresponding receptors. Overall, these three studies are still insufficient due to their weaknesses and the inhomogeneity of the results. A comprehensive study that does not focus solely on one BB with a nonspecific effect would be helpful. In addition, more parameters should be measured in one experimental set.

The study by Al-Subaie presents the results in a more multi-layered way by looking at both micro-CT and histology [73]. A disadvantage is the use of implants that have only a machined surface and are not comparable to commercially available products. This surface treatment might even have an osteoinductive effect [123].

The effects presented are comparable to results found for BMP-2, which showed an improvement in osseointegration of almost 50% [124,125]. These studies do not agree in all aspects, but it is impressive as BMP-2 has received much attention in the literature [126,127]. Compared to propranolol, BMP-2 has crucial disadvantages for future use. These include high costs, the likelihood of immune reactions and even cancer promotion [128,129,130,131]. Propranolol, on the other hand, has a positive effect on the survival of these patients [132,133].

The change in osteoclast numbers due to the suppression of RANKL activation is consistent with other studies [94,134]. In addition, propranolol increases collagen synthesis, a component of new bone formation and stability, via a cAMP-dependent signaling pathway [135]. Rodrigues and Bonnett showed that even low doses of propranolol are sufficient to affect bone metabolism. Thus, no cardiovascular side effects occur, and future clinical applications are less risky [134,136].

As in the study by Tekin et al., the rats were still growing at the age of about 2.5 months. Al-Subaie concluded, based on a publication by Aguirre, that they respond less to drugs affecting the bone [137].

At first glance, one would like to think that Zhou’s study contradicts the work on propranolol [74]. However, norepinephrine has a significantly higher affinity for β1-receptors [138]. As mentioned previously, it is mainly the stimulation of β2 that leads to bone resorption. As Gille et al. already showed in 1985, the blocking effect of propranolol on beta 2 is about three times higher than on β1 [139]. Thus, a correlation can be observed here as well.

It would be reasonable to assume that the electrical stimulation could lead directly to altered bone conditions. On the contrary, the electrodes were located far from the actual implantation site. On the other hand, studies by Dergin showed that electrical stimulation alone had no effect. Thus, it becomes more likely that the SNS is especially relevant [140,141].

The cell culture results should be interpreted with caution. It is rather unphysiological that the cell bodies grow directly adjacent, although direct contact of all bone cells to sympathetic fibers could be demonstrated [30,89]. Stimulation via NE released into the environment is also possible [80]. Investigations in co-culture may reveal new mechanisms: He et al. cultured osteoblasts with sympathetic neurons and found an increased synthesis of BMP by the neurons. Thus, the SNS could also influence bone metabolism independently of ARs [142].

Yao et al. used a chemical sympathectomy to eliminate the sympathetic nervous system [75]. This method is well-established [143]. Since the substance cannot cross the blood–brain barrier, only the peripheral sympathetic nervous system is affected. Accordingly, there is no release of NE or its cotransmitters in treated rats [144]. The effect on osteointegration contrasts with the results of studies on propranolol and in the field of orthopaedics. Cherruau et al. used guanethidine, and by inhibiting osteoclasts, bone resorption was stopped [145]. Chemical sympathectomy is not a possible option for humans and reduces the clinical relevance. The direct influence of the chemicals used for sympathectomy is also hard to quantify. Future studies should also investigate the specific effect of NE on bone cells compared to the in vivo effect, because an altered blood supply might influence it [29]. The specific inhibition of receptors might be superior to complete SNS inhibition, including cotransmitters and circulating epinephrine.

### 4.3. Future Perspectives

The present review aimed to shed light on the effect of the sympathetic nervous system in dental implantology and to discuss its relevance for future research. Despite the partially heterogeneous results, this topic holds a non-negligible potential. The goal for the future is to bridge the gap between laboratory and clinical applications.

Clinical studies showed that antidepressants that increase norepinephrine concentrations reduce implant success, while beta-blocker therapy failed to show any differences in implant stability [66,67]. These were retrospective analyses, which are relevant, but the derivation of causal relationships is not possible [146]. Furthermore, the differentiation of the mentioned drugs is still too superficial, and the number of cases is far too small. Studies with substances that specifically act on the receptors are needed. Preclinical studies should use implants as close to reality as possible.

Should an effect be confirmed, imaginable applications would be systemic therapy and a local application of sympathetically active substances. Thus, oriented to work on BMP, the drugs identified as most effective could be applied to implant surfaces [147], furthermore enriched in bone substitutes [148] or incorporated into membranes [149]. Delayed-release drug-delivery systems are available and would be an additional application [150]. Propranolol is a drug that has been established for many decades with calculable side effects, especially at low doses [134]. It inhibits cancer cell migration and tumor invasion in oral squamous cell carcinoma [151]. A protective effect would be possible in contrast to BMP. Rehabilitation by implants after tumor surgery is also a topic of current research [152].

Song et al. compared the fracture risk of elderly patients under antihypertensive therapy in a study with over 500,000 patients and found no protective effect of unselective BB. For β1-selective BB users, however, the fracture risk was about 35% lower [153]. Possibly patients with limited bone metabolism might profit from additional treatment. Since implants already show very high success rates, it may be more of a goal in the future to expand the range of indications and allow more patients to be treated with dental implants, regardless of their pre-existing conditions [154,155].

But not only the therapeutic application could be deduced: an overactive sympathetic nervous system could be a risk factor. The study by Hakam et al. already showed that antidepressants acting on NE release might be of relevance for implant survival [67]. A recent publication about hip prostheses revealed a reduced loosening risk with BB therapy [156].

The so-called human factors are gaining importance throughout medicine [25,157,158,159]. One integral part of this concept is stress reduction, not only for the practitioner, but also for the patient [25,160]. Since the sympathetic nervous system is involved in processing stress, this aspect should also be considered [84]. There is a possibility that, mediated in part by the SNS, limitations in wound healing occur due to impaired angiogenesis, and ARs also appear to be involved [161,162]. Effects of human factors interventions could thus even be explained and measured by physiological mechanisms, which has been one of the main problems in this field so far [163].

As a final point, the focus of this review was mainly on bone and osseointegration due to the results in the literature. The soft tissue, which is essential for successful implant therapy [164,165] and also supplied by sympathetic nerve fibers, has not been considered yet.

## 5. Conclusions

Overall, the sympathetic nervous system offers potential for dental implantology, especially since most current studies lack sufficient quality. Therefore, the first two questions of this review can be discussed, but not finally answered right now. Publications from other specialties mention the influence on bone metabolism, and there seem to be complex interactions between NE, its cotransmitters and the systemic effect of the SNS. NE might have a catabolic effect on bone cells, while the findings on sympathectomy and SNS activation are not consistent [74,75,116,145]. Specific targeting and understanding of the interactions with implant surfaces will be vital. Only after evaluating the underlying mechanisms (question three) is the development of new therapies possible and a wide range of applications realistically feasible.

Medication and its influence on dental implants are highly relevant, but the data are insufficient to provide clinical recommendations for sympathomimetic and sympatholytic drugs. Nevertheless, basic research provides the first approach to answering the fourth question.

The fascination with influencing the remodeling of an amazingly dynamic tissue such as bone via the nervous system [166] will hopefully lead to further insights and the development of new therapies and clinical guidelines.

## Figures and Tables

**Table 1:** Search terms. The asterisk (*) is the symbol for truncation.

Autonomous Nervous System		Dental Implantology
Adrenergic * OR Adrenoceptor * OR Norepinephrin * OR sympathetic nerv * OR autonomic nervous system OR parasympathetic nerv *	AND	maxillofacial surge * OR oral surge * OR dental surge * OR operative dentist * OR oral implant * OR dental implant * OR peri implant * OR periimplant *

**Table 2:** Preclinical studies.

	Test-Subject	Implant (Type and Localization)	Intervention	Outcome-Parameter
Morinaga et al. (2019) [68]	Mouse primary BMSCs	The cells were cultured on a polystyrene plate, sandblasted and acid-etched titanium discs or machined titanium discs		**Chemical genetics study** Identification of relevant signaling pathways. Under the following citation, a detailed description of the process of chemical genetics analysis is given [77] **Expression of ARs** RTPCR, mRNA levels of α1a, α1b, α1d, α2a, α2b, α2c, β1, β2 and β3 adrenergic receptors
Tavakoli et al. (2022) [69]	Four nondomestic male street dogs	Bone level implants (SNUCON, Korea), 4 mm in diameter and 10 mm in length Second, third, and fourth premolar in the left mandible	Extraction of three teeth Test: propranolol oral tablet 0.2 mg/kg, Control: saline Three implants after 2 months, submerged healing After 4 and 9 weeks, dental implants and the peripheral bone were removed using a 6-mm trephine drill	**Histological analysis** Bone implant contact (BIC)
Yildirim et al. (2021) [70]	20 Sprague–Dawley rats	Machined-surfaced titanium implants, 4 mm in length, with a diameter of 2.5 mm (Implance Dental Implant System, AGS Medical, Istanbul, Turkey) Metaphyseal part of each tibia	Insertion of the implant Test: 10 mg/kg propranolol orally on every day for 4 weeks Control: No further treatment	**Blood sample analysis** Alkaline phosphatase, calcium, phosphorus **Histological analysis** Bone implant connection (BIC)
Tekin et al. (2021) [71]	24 Sprague–Dawley rats	Machined-surfaced titanium implants, 4 mm in length, with a diameter of 2.5 mm (Implance Dental Implant System, AGS Medical, Istanbul, Turkey) Metaphyseal part of each tibia	Implant insertion Three groups for the 4-week experiment: (1) No further treatment (2) 5 mg/kg propranolol orally 3 days a week (3) 10 mg/kg propranolol orally 3 days a week	**Blood sample analysis** Alkaline phosphatase, calcium, phosphorus **Biomechanical analysis** Reverse torque test
Gunes et al. (2021) [72]	24 Sprague–Dawley rats	Resorbable blast material titanium implants, 2.5 mm diameter and 4 mm in length with eight threads (AGS Medical Corporation; Istanbul, Turkey) Metaphyseal part of each tibia	After implant insertion, a three-walled standard defect of 2.5 mm width and 2 mm length was opened, hydroxyapatite bovine bone graft was placed in the defect Three groups for the 8-week experiment: (1) No further treatment (2) 5 mg/kg propranolol orally 3 days a week (3) 10 mg/kg propranolol orally 3 days a week	**Blood sample analysis** Alkaline phosphatase, calcium, creatinine, phosphorus **Histological analysis** Newly formed bone area New bone formation rate
Al-Subaie et al. (2016) [73]	24 Sprague–Dawley female rats	Cylindrical cuts of a titanium rod, 1.5 mm in diameter and 2 mm in length Metaphyseal part of each tibia	One side: hole with 1.5 mm in diameter Contralateral: hole with 2.5 mm in diameter Test group: 5 mg/kg propranolol, subcutaneous, daily for 2 weeks Control: saline	**Microcomputed tomography** Cortical defect volume, bone volume/tissue volume, trabecular thickness, trabecular number, and trabecular separation **Histology of the bone defects** Osteoclast number per square millimeter of mineralized tissue, mineralized tissue percentage and collagen percentage **Histology of the bone implant contact** Bone implant contact measurements (total, cortical and medullary), cortical and medullary peri-implant bone volume/tissue volume (BV/TV)
Zhou et al. (2019) [74]	Eight female beagles	Pure titanium self-produced machined implants 4.0 mm in diameter 7.0 mm in length Maxillary lateral incisors	Immediate implantation Three double-implant beagles and one single-implant beagle for each group 1 week after implantation: test group electrically stimulated transcutaneously for 45 min each day for 3 weeks Control group: no stimulation	**Microelectrodes bilaterally in the infraorbital nerves** Electric potential of the sympathetic nerve fibers in the infraorbital nerve **ECG monitor** Blood oxygen saturation and heart rate **Microcomputed tomography analysis** Bone volume percentage, trabecular thickness, trabecular number, trabecular separation **Histological analysis** Morphological analysis
Yao et al. (2019) [75]	40 C57BL/6J mice	Rod-shaped machined titanium implants, 3 mm in length and 1 mm in diameter in the anterior-distal surfaces of both femurs in each mouse	Test group: sympathectomy with 6-hydroxydopamine 5 days before surgery Implant placement One femur from each mouse was harvested at week 2 and 4 after surgery	**Microcomputed tomography (micro-CT)** Bone volume to total volume ratio, bone surface to total bone volume ratio, mean trabecular number, mean trabecular thickness, mean trabecular separation, percentage of osseointegration **Blood sample analysis** Osteocalcin and C-terminal collagen I cross-links **Histological analysis** Mineral apposition rate, bone formation rate per bone surface, bone-to-implant contact, number of osteoclasts per peri-implant surface **Biomechanical analysis** Push-in test

## Data Availability

Data sharing is not applicable to this article.
